# Cross-Sectional Associations of Total Daily Volume and Activity Patterns across the Activity Spectrum with Cardiometabolic Risk Factors in Children and Adolescents

**DOI:** 10.3390/ijerph17124286

**Published:** 2020-06-16

**Authors:** Simone J. J. M. Verswijveren, Karen E. Lamb, Anna Timperio, Jo Salmon, Rohan M. Telford, Robin M. Daly, Ester Cerin, Clare Hume, Lisa S. Olive, Kelly A. Mackintosh, Melitta A. McNarry, Nicola D. Ridgers

**Affiliations:** 1 Institute for Physical Activity and Nutrition, School of Exercise and Nutrition Sciences, Deakin University, Geelong 3220, Australia; klamb@unimelb.edu.au (K.E.L.); anna.timperio@deakin.edu.au (A.T.); jo.salmon@deakin.edu.au (J.S.); robin.daly@deakin.edu.au (R.M.D.); nicky.ridgers@deakin.edu.au (N.D.R.); 2 Murdoch Children’s Research Institute, Royal Children’s Hospital, Parkville 3052, Australia; 3 Department of Paediatrics, University of Melbourne, Royal Children’s Hospital, Parkville 3010, Australia; 4 Melbourne School of Global and Population Health, Centre for Epidemiology and Biostatistics, Melbourne School of Population and Global Health, University of Melbourne, Carlton 3053, Australia; 5 Research Institute of Sport and Exercise, University of Canberra, Bruce 2601, Australia; rohan.telford@canberra.edu.au; 6 Mary MacKillop Institute for Health Research, Australian Catholic University, Melbourne 3000, Australia; ester.cerin@acu.edu.au; 7 School of Public Health, The University of Hong Kong, Hong Kong 749H+FP, China; 8 School of Public Health, University of Adelaide, Adelaide 5000, Australia; clare.hume@adelaide.edu.au; 9 School of Psychology, Deakin University, Burwood 3125, Australia; lisa.olive@deakin.edu.au; 10 Institute for Mental and Physical Health and Clinical Translation, Deakin University, Geelong 3220, Australia; 11 Australian National University Medical School, Australian National University, Garran 2605, Australia; 12 Applied Sports, Technology, Exercise and Medicine Research Centre, Swansea University, Swansea SA1 8EN, UK; k.mackintosh@swansea.ac.uk (K.A.M.); m.mcnarry@swansea.ac.uk (M.A.M.)

**Keywords:** physical activity, sedentary behavior, accumulation patterns, child, adolescent, cardiometabolic health

## Abstract

Sedentary and physical activity patterns (bouts/breaks) may be important for cardiometabolic health in early life. This study aimed to examine cross-sectional associations of total daily volume and patterns across the activity spectrum with cardiometabolic risk factors in youth aged 7–13 years. Objectively measured accelerometer and cardiometabolic risk factor data were pooled from two studies (*n* = 1219; 69% valid accelerometry). Total daily volume of sedentary time and light-, moderate-, and vigorous-intensity physical activity was determined. Time in sustained bouts and median bout lengths of all intensities and breaks in sedentary time were also calculated. Outcomes included body mass index, waist circumference, blood pressure, blood lipids, and a cardiometabolic summary score. Regression models revealed beneficial associations between total daily volumes of moderate- and vigorous-intensity physical activity and cardiometabolic risk. Time spent in ≥1 min vigorous-intensity physical activity bouts was beneficially associated with cardiometabolic risk, yet this disappeared after adjusting for total vigorous-intensity physical activity and confounders. Time accumulated in light- (≥1 min; ≥5 min) and moderate-intensity (≥1 min) physical activity bouts was detrimentally associated with cardiometabolic risk. Total daily volume and activity patterns may have implications for cardiometabolic risk early in life. Sporadic physical activity may be more beneficial for health than sustained physical activity.

## 1. Introduction

Low physical activity (PA) and high sedentary (SED) behavior engagement are associated with precursors for poor cardiometabolic health in youth [1,2]. Little research has examined whether the duration and frequency of bouts and breaks in PA and SED time (from here on “activity patterns”) are associated with health risk factors compared to total daily volumes [3]. In adults, research has shown that activity patterns, including both SED and PA accumulation, such as prolonged ≥10 min PA bouts and breaks in sitting time, are important for cardiometabolic risk, including reduced adiposity and improved glucose metabolism [4]. As cardiometabolic risk factors and activity levels tend to track from child- into adulthood [5,6], a detailed understanding of activity patterns and their association with cardiometabolic health among young people is needed.

To date, activity pattern research in youth has primarily examined adiposity indicators as a precursor for the development of cardiometabolic disease, ignoring potential associations with other cardiometabolic risk indicators, such as blood lipids and blood pressure [3]. Obesity during childhood is associated with increased blood pressure and lipids, which in turn can lead to increased cardiometabolic risk later in life [6,7]. The reliance of published studies on adiposity indicators only is a major weakness in evidence to date. As such, activity pattern research that explores alternative risk factors is required to inform future interventions aimed at improving youth cardiometabolic health. This study aimed to examine associations of total daily volume and patterns across the activity spectrum with cardiometabolic risk factors in youth aged 7–13 years.

## 2. Materials and Methods

### 2.1. Participants

Accelerometer and health data were drawn from two trials: “Lifestyle of Our Kids” (LOOK; trial registration: ACTRN12615000066583 [23/01/2015] [8]) and “Transform-Us!” (ACTRN12609000715279 [19/08/2009], ISRCTN83725066 [30/06/2010] [9]). Ethical approval was provided by the Australian Capital Territory Health Human Research Ethics Committee (approval number: ETH.9/05.687) and Deakin University Human Research Ethics Committee (EC2009-141), respectively. Details of the individual trials are reported in earlier publications [8,9,10]. Parental consent was collected prior to data collection (*n* = 1452; 59% LOOK). Baseline data from Transform-Us! (2010) and data from time-point five [10] from LOOK (2009; first test occasion that included both accelerometry and blood collection) were used. In total, 1219 participants provided raw data for at least one assessed variable in this study (52% LOOK). The participant flow diagram is presented in Figure S1.

### 2.2. Measures

#### 2.2.1. Accelerometry

Participants were fitted with an ActiGraph accelerometer (ActiGraph, Pensacola, Florida, United States) and asked to wear this on their right hip for at least seven consecutive days. LOOK and Transform-Us! participants wore the GT1M [10] model and the GT3X [9] model, respectively; these have acceptable comparability [11]. The ActiLife software (v5.1.5; www.actigraphcorp.com) was used to convert raw data into 15 s epoch files afore using a customized Microsoft Excel macro to obtain the accelerometry exposure variables. Non-wear time was defined as ≥20 min of consecutive zeroes [12]. A valid day was defined as ≥8 h of wear-time on weekdays, and ≥7 h of wear-time on weekend-days [12]. The lower weekend wear-time threshold was set to account for later waking time compared to weekdays. Participants with ≥4 complete days were included for analyses [12]. The SED threshold was set as <100 counts/min [13]. Freedson cut-points were used to determine total daily volume and activity patterns of light- (LPA; ≥100 counts/min and <4 metabolic equivalent of tasks (METs)), moderate- (MPA; ≥4 and <6 METs), and vigorous-intensity (VPA; ≥6 METs) PA [14]. These cut-points were previously developed in a laboratory study where 6–18 year olds engaged in different walking and running speeds whilst respiratory gas exchange was measured using indirect calorimetry and an ActiGraph accelerometer was worn [14].

In accordance with previous studies [3], bouts were defined as sustained periods of ≥5 and ≥10 min of SED time and ≥1, ≥5, and ≥10 min of time in LPA, MPA, and VPA. These durations are considered prolonged behaviors as children typically accumulate their activity sporadically, particularly compared to adults (e.g., 80% of their high-intensity activities last less than 10 consecutive seconds [15]). No tolerance (i.e., interruption in intensity) was allowed in defining bouts, based on previous recommendations for SED [16] and in the absence of recommendations for PA bouts. Breaks in SED time were defined as where a non-SED epoch interrupted SED time [17]. Finally, median bout durations for all intensities were determined by calculating all uninterrupted bouts within the same intensity starting from the shortest bout (i.e., ≥1 min bouts for PA; ≥5 min bouts for SED) to the longest bout and then identifying the mid-point [18]. Mean values for all accelerometry variables were calculated over all valid days.

#### 2.2.2. Cardiometabolic Risk Factors

Seven cardiometabolic risk factors were objectively assessed: body mass index (BMI), waist circumference (WC), systolic (SBP) and diastolic blood pressure (DBP), high-density (HDL-C) and low-density (LDL-C) lipoprotein cholesterol, and triglycerides (TG). Height, weight, and WC were measured using standardized procedures [19]. BMI was calculated and converted to the World Health Organization (WHO) Child Growth Standards age- and sex-standardized z-values (zBMI) [20]. Subsets of children provided blood pressure and/or lipid data (see Figure S1), which were collected via standardized procedures (i.e., seated; fasted venous blood samples [9,10]). Data from both studies were combined and standardized z-values for each measure were calculated (z-value = [value − mean]/SD). As HDL-C is inversely related to cardiometabolic risk it was multiplied by −1. Then, a continuous cardiometabolic summary score (CMR-score) was derived by summing the z-values of WC, SBP, DBP, LDL-C, HDL-C, and TG; higher scores indicated higher cardiometabolic risk [16]. 

#### 2.2.3. Participant Characteristics

Participant characteristics included age, sex, and area-level socioeconomic status (SES). Age and sex were self-reported by the participants and their parents. Postal codes for school locations were classified as low (i.e., first quintile), mid (i.e., second–fourth quintile), and high (i.e., fifth quintile) SES using the national Index of Relative Socioeconomic Disadvantage of the Socioeconomic Indexes Areas Score [21]. Trial involvement (LOOK, Transform-Us!), sex, and SES were used as categorical variables; age was used as a continuous variable. In addition, school was identified as a cluster-level characteristic.

### 2.3. Statistical Analyses

Analyses were performed using Stata Version 15.0 (StataCorp, College Station, TX, USA). To be included in analyses, participants had to have at least valid accelerometry, and complete adiposity indicators and confounder data (‘included sample’). Data inspection showed that some predictor variables were highly zero-inflated, meaning that only low proportions of participants engaged in those specific activity patterns, and, thus, these variables were excluded from analyses. A subset of participants additionally had blood pressure and/or lipids data (see Figure S1). The CMR-score analysis required participants to have complete data for all cardiometabolic risk factors. To check the assumption that data were missing completely at random, characteristics of the included and excluded samples were compared. 

Linear regression models were fitted to obtain β regression coefficients and 95% confidence intervals (CIs) for the associations between each activity exposure (i.e., total daily volumes and activity patterns) and each cardiometabolic risk factor. Models included cluster-robust standard errors to account for clustering within schools. Minimally adjusted and multiple adjusted models were considered to assess the effect of covariate adjustment on the association of interest. For total daily volume exposures, Model 1 only adjusted for wear-time and trial involvement (minimally adjusted). Model 2 additionally adjusted for age and sex (partially adjusted), and Model 3 further adjusted for SES (fullyadjusted). Similar models were fitted for activity pattern exposures, however, partially adjusted Model 2 included adjustment for the total daily volume of the corresponding intensity (e.g., total LPA for LPA bouts) in addition to minimally adjusted Model 1. This enabled the examination of associations between activity patterns and cardiometabolic health, regardless of the total daily volume of the intensity. Models 3 and 4 subsequently adjusted for the same covariates as described in Model 2 and 3 for total daily volumes. Adiposity was not included as a covariate in models of non-weight-related cardiometabolic risk factors as it may mediate associations between activity patterns and cardiometabolic health outcomes [22]. 

Wear-time was highly correlated (r ≥ 0.80) with the total daily volume of SED, and SED breaks ans bouts. Thus, total SED time was adjusted for wear-time using residuals obtained by regressing total daily volume of SED on wear-time [13]. Time spent in ≥5 and ≥10 min SED bouts, and the frequency of SED breaks, were adjusted for total wear-time and total daily volume of SED using this method [13]. This method makes highly correlated variables perfectly uncorrelated and is commonly used in behavioral research to manage collinearity issues [13]. No other variables that were assessed within the same regression models were highly correlated (r ≥ 0.80) or showed potential for collinearity issues (variance inflation factor ≥ 10); data not presented. All other assumptions for linear regression were met. 

## 3. Results

### 3.1. Sample Characteristics

Participant characteristics per trial (i.e., LOOK and Transform-Us!) are reported in Table S1. Participants in Transform-Us! were younger compared to LOOK participants. Valid accelerometer data were obtained from 69% of participants (843/1219). Of those participants, 93% (*n* = 782; 64% of the originally obtained participants) had complete adiposity indicators and confounder data (‘included sample’); 76% (*n* = 637) had complete data to be included in the blood pressure subset; 62% (*n* = 525) for the lipid subset, and 48% (*n* = 404) for the CMR-score subset (see Figure S1). Lower numbers in the subsets were mostly due to a lack of consent for these assessments. Characteristics of included and excluded samples were largely comparable (Table S2).

### 3.2. Accelerometer-Derived Variables

Table S3 presents the accelerometer-derived variables for included participants (i.e., those with valid accelerometry, adiposity, and confounder data). On average, participants spent 60% of their time SED and 31%, 6%, and 3% of their time in LPA, MPA and VPA, respectively. More time was accumulated in shorter bouts than in longer bouts, regardless of intensity. Whilst pattern variables were defined *a priori* based on previous research [3], five of the pattern variables were only engaged in by a quarter of the sample or less and, thus, excluded from further analyses (see Table S3). Consequently, seven specific patterns variables remained included: the number of breaks in SED, and the time spent in ≥5 min (39% of total daily volume of SED) and ≥10 min SED bouts (19%), ≥1 min (45% of total daily volume of LPA) and ≥5 min LPA bouts (1%), ≥1 min MPA bouts (19% of total daily volume of MPA), and ≥1 min VPA bouts (28% of total daily volume of VPA).

### 3.3. Associations between Total Daily Volumes and Cardiometabolic Risk Factors

Table 1 shows associations between total daily volumes and cardiometabolic risk factors. Minimally adjusted Model 1 and fully adjusted Model 3 are presented [23]. Findings were consistent across the minimally adjusted Model 1, partially adjusted Model 2, and fully adjusted Model 3, meaning that direction and significance did not change, and effect sizes stayed comparable after stepwise adjusting for potential confounders. Therefore, the partially adjusted Model 2 is only reported in Table S4. Total daily SED volume was significantly detrimentally associated with TG levels only. Total daily volume of MPA and VPA were significantly beneficially associated with HDL-C, TG, and CMR-score. Total daily volume of VPA was additionally significantly beneficially associated with zBMI, WC, and LDL-C. For all outcomes, the effect sizes obtained for VPA were larger than the effect sizes obtained for MPA. Total daily volume of LPA was not significantly associated with cardiometabolic risk factors.

### 3.4. Associations between Activity Patterns and Cardiometabolic Risk Factors

**Figure 1:**
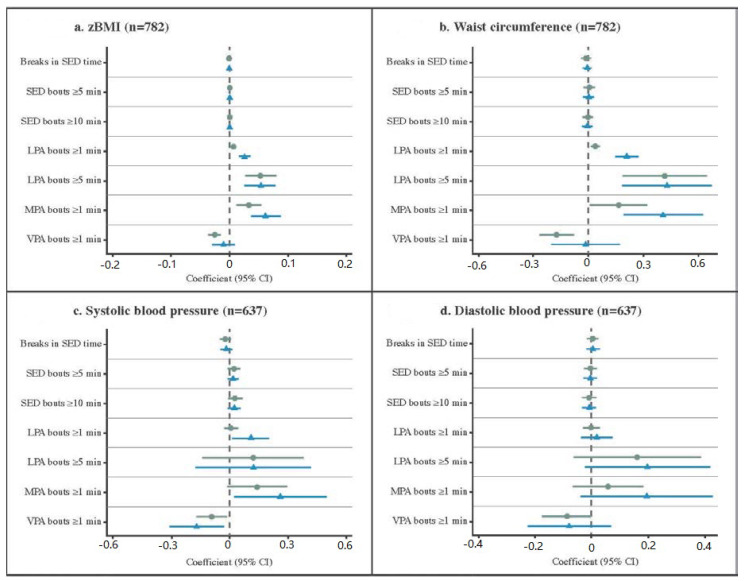

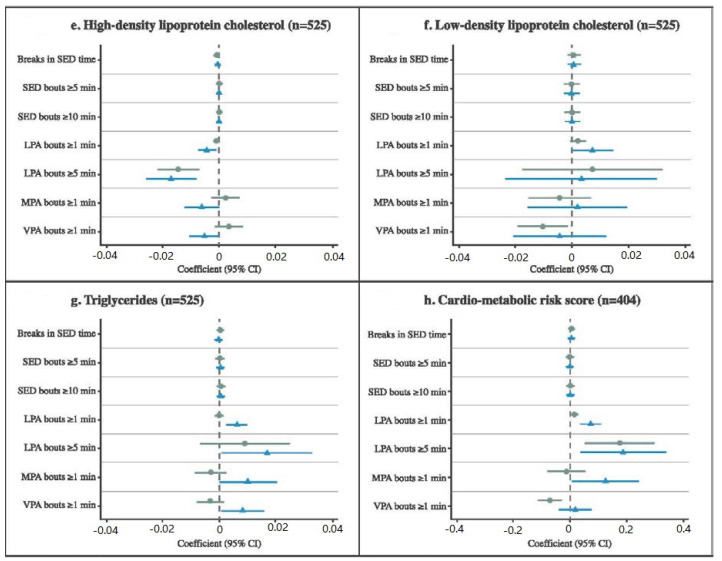
Associations of activity patterns with cardiometabolic risk factors. Coefficient (95% CI): regression coefficients and 95% confidence intervals. Coefficients and 95% Cis were standardized across figures to aid comparison between variables. ● Model 1 (adjusted for wear-time and trial involvement, and accounted for clustering within schools). ▲ Model 4 (further adjusted for the total daily volume of the corresponding intensity, participants’ age, sex, and socioeconomic status). Results from the partially adjusted Model 2 (included adjustment for the total daily volume of the corresponding intensity in addition to minimally adjusted Model 1) and 3 (additionally adjusted for age and sex in addition to partially adjusted Model 2) can be found in Tables S5 and S6. Breaks in SED (number per day) were defined as an interruption in SED where counts exceeded 25 counts per 15 s epoch [4,17] and averaged over all valid days. Bouts were calculated as the daily time accumulated in either ≥1, ≥5, or ≥10 min bouts of the corresponding intensities (in minutes) and averaged over included days. SED: sedentary; LPA: light-intensity physical activity; MPA: moderate-intensity physical activity; VPA: vigorous-intensity physical activity; zBMI: body mass index converted to the World Health Organization (WHO) Child Growth Standards age- and sex-standardized z-values [20]; min: minutes. Figure 1a. zBMI (*n* = 782); Figure 1b. Waist circumference (*n* = 782); Figure 1c. Systolic blood pressure (*n* = 637); Figure 1d. Diastolic blood pressure (*n* = 637); Figure 1e. High-density lipoprotein cholesterol (*n* = 525); Figure 1f. Low-density lipoprotein cholesterol (*n* = 525); Figure 1g. Triglycerides (*n* = 525); Figure 1h. Cardiometabolic risk score (*n* = 404).

Figure 1 shows associations of bouts and breaks with cardiometabolic risk factors from the minimally adjusted Model 1 and fully adjusted Model 4. The β-coefficients and 95% confidence intervals for all models, including partially adjusted Models 2 and 3, are reported in Tables S5 and S6. To aid readability, only the statistically significant (*p* < 0.05) results for the fully adjusted Model 4 are reported in the following text. Time in ≥1 min LPA and ≥5 min LPA bouts was detrimentally associated with zBMI, WC, HDL-C, TG, and CMR-score. In addition, the time in ≥1 min LPA bouts was detrimentally associated with SBP and LDL-C. Time in ≥1 min MPA bouts was detrimentally associated with zBMI, WC, SBP, TG, and CMR-score. Whereas time spent in ≥1 min bouts of VPA was beneficially associated with SBP, it was detrimentally associated with levels of TG. There was no evidence of an association between SED patterns (i.e., bouts and breaks) and cardiometabolic risk factors. 

Overall, consistent detrimental associations were observed for time spent in ≥1 min LPA bouts, ≥5 min LPA bouts, and ≥1 min MPA bouts with cardiometabolic risk factors; no consistent beneficial associations were found. The effect sizes obtained for time in ≥5 min LPA bouts were similar to the effect sizes obtained for time in ≥1 min MPA bouts; both were larger than those for time in ≥1 min LPA bouts. For example, after accounting for covariates and total time in their intensities, an additional minute of time in ≥1 min MPA bouts, ≥5 min LPA bouts, and ≥1 min LPA bouts, was associated with 0.41, 0.43, and 0.21 cm higher WC, respectively. This corresponds to 12.26, 12.87, and 6.29 cm higher WC for every additional 30 min/day spent in ≥1 min MPA bouts, ≥5 min LPA bouts, and ≥1 min LPA bouts, respectively. 

Associations between median bout lengths and cardiometabolic risk factors from minimally adjusted Model 1 and fully adjusted Model 4 are reported in Figure S2. Specific results for all models are reported in Tables S5 and S6. To aid readability, statistically significant findings for fully adjusted Model 4 are discussed in the following text only. A higher median LPA bout length was detrimentally associated with zBMI, WC, and HDL-C. In other words, a higher usual LPA bout duration was associated with poorer cardiometabolic risk factors. No other evidence for significant associations was found.

## 4. Discussion

This study examined associations between total daily volume and activity patterns across the activity spectrum and cardiometabolic risk factors in youth. Consistent with previous evidence, the total accumulated total daily volumes of MPA and VPA were beneficially associated with cardiometabolic risk [2]. Total daily volume of SED was detrimentally associated with TG, yet no other associations with cardiometabolic risk factors were observed. This is consistent with a systematic review that concluded that it is difficult to observe associations between objectively measured total daily SED time and health in observational studies [1]. Interestingly, no associations were found between total daily volumes of LPA with cardiometabolic risk in this sample, which contrasts previous research that has shown beneficial associations between LPA and cardiometabolic health in this age group [13].

With regards to the activity patterns, higher time in ≥1 min VPA bouts was beneficially associated with cardiometabolic risk, however, the significance (using *p* < 0.05) of the association disappeared after adjusting for total VPA for most of the cardiometabolic risk factors (Tables S5 and S6). Although the coefficients remained of similar size and direction, this suggests that the total volume of VPA, rather than the way in which it is accumulated, might be most important and that health benefits in youth may not have to include prolonged (i.e., ≥1 min) engagement in high-intensity activities. These results are consistent with previous research in this age group [24] and are useful for the design of interventions. VPA of any duration should be promoted as effective and time-efficient options to improve health [24].

Whilst total daily volume of MPA was beneficially associated with cardiometabolic health, detrimental associations between MPA patterns and cardiometabolic risk factors were found. Whilst our findings need further verification, this suggests that MPA accumulated sporadically (i.e., <1 min) instead of in longer bouts (i.e., ≥1 min) may benefit health. This supports previous research that found that substituting short bouts of high-intensity activity with the same amount of activity accumulated in long bouts was associated with a higher (i.e., worse) cardiometabolic risk score [24], and research that found that short intermittent bursts of PA were favorably and more strongly associated with children’s metabolic health than longer bouts [25]. However, these findings are contrary to some research showing that ≥MPA-intensity activity, regardless of duration, is beneficial to health [2]. Perhaps time spent in prolonged MPA co-occurs with unhealthy behaviors in children (e.g., resulting in lower levels of VPA across the day; increased sitting time and/or poorer diet after sustained activity [26]), which may cause the observed detrimental impact. Future research needs to focus on co-occurring behaviors for these activity patterns. Most international guidelines state that youth should accumulate ≥60 min of moderate-to-vigorous-intensity PA per day, yet do not recommend how this should be accumulated [27]. As it may be easier to implement short MPA bursts than longer bouts into a child’s day, further research needs to examine the cardiometabolic benefits associated with MPA accumulated in intermittent versus continuous patterns. This, in turn, can inform future guidelines and interventions on the inclusion of potential PA accumulation recommendations and strategies.

Whilst total daily volume of LPA was not associated with cardiometabolic risk factors, there was consistent evidence across models of detrimental associations between LPA bouts and cardiometabolic risk, especially long LPA bouts and cardiometabolic health. Whilst it is assumed that any activity, regardless of intensity, is beneficial to health [2], our finding has some support in the existing literature in relation to adiposity [28]. There are several possible explanations for the detrimental associations observed for sustained LPA bouts. Previous evidence, albeit in adults, suggests that static (i.e., standing) LPA is less beneficial to health compared to dynamic LPA, and that prolonged standing bouts might be detrimental for cardiometabolic health [29]. The recorded bouts in the current study may have included prolonged static LPA and/or sitting (cut-points used to classify activities did not have the ability to discern sitting from standing), causing detrimental effects on health. Alternatively, participants who accumulated the most time in LPA bouts may have spent less time in higher intensities. For example, these children may have spent recess and lunch mostly in LPA bouts, as opposed to engaging in higher intensities. As waking time is finite, the time spent in these behaviors is co-dependent [30], and thus the detrimental associations of LPA bouts may have reflected a lack of high-intensity activity. Further research including statistically advanced analytical methods such as compositional analysis [30] is required to elucidate the potential mechanisms behind these associations. Research exploring the types of behaviors that children are participating in would also shed further light on such mechanisms. 

### 4.1. Defining Activity Patterns

Most youth in this sample did not engage in ≥5 and ≥10 min bouts MPA or VPA bouts; therefore, these variables were excluded from analyses. Whilst these bouts are commonly used patterns in previous research [3], our data suggest that these are uncommon. This may be explained by the decision to allow no tolerance in the bout definition but also highlights the sporadic nature of youth activity accumulation [15]. Arguably, ≥1 min PA bouts happen so infrequently that minimal health benefits may be gained and therefore interventions might be best focused on increasing sporadic PA across the day. There is a need to investigate different types of activity patterns among youth and their potential impacts on health. In this study, the results for median bout durations of all intensities are consistent with presented associations observed for the specified bout durations. As some of the longer bouts were rarely observed, median bout duration might be a more appropriate variable for future studies as it reflects typical patterns of behavior and aids comparisons between studies. 

### 4.2. Strengths and Limitations

The strengths of this study include the examination of associations between objectively measured activity patterns across the activity spectrum and a range of objectively measured cardiometabolic risk factors in a large sample of youth with a wide age-range. However, several limitations should be acknowledged. Firstly, chronological age is not a strong indicator by which to assume maturity stage. As maturity may influence cardiometabolic risk factors in adolescents, future studies will need to incorporate this characteristic. This sample included relatively active and healthy youth, which may have attenuated potential evidence of associations; whether these findings persist in a higher risk cohort remains to be evaluated. Although the LOOK trial did not target activity patterns (i.e., breaking up sitting) and found no intervention effect on school week PA levels, it is unclear whether the intervention may have influenced our findings. The waist-worn accelerometers and cut-points used to classify activities did not have the ability to discern sitting from standing. There is thus a possibility that LPA bouts were indicative of some SED [14]. While such methods have been shown to have acceptable validity for assessing total daily volume of SED, these could misclassify sitting as standing, and thus as LPA. Consequently, research using posture-based measures, such as activPAL (www.palt.com), are needed to more accurately distinguish LPA and SED patterns. 

There are also statistical limitations that need to be acknowledged. Firstly, due to the high number of results, there may be an increased likelihood of false discovery due to multiple testing. Secondly, in order to compare the patterns observed in our study with those in previously conducted studies [3], and to identify which activity patterns are important for health, individual regression models between activity patterns and cardiometabolic risk factors were conducted. This approach, involving single-intensity patterns within traditional regression models, has been widely used in the literature to date and is arguably still the most commonly used methodology to assess associations between bouts/breaks and cardiometabolic risk factors [3]. The adjustment for total volumes in Model 2 is imperfect as the time in bouts are inherently related to (part of) the total time in their intensity, and this may have led to collinearity issues. The decision was therefore made to present the models stepwise (i.e., with and without total volume adjustments), and although the direction and effect sizes did not substantially change from these specific adjustments, results should be interpreted cautiously. Future studies should consider using statistically advanced analytical methods (e.g., compositional analysis [30], multivariate pattern analysis [25]) to verify our findings. Finally, due to the cross-sectional nature of this study, it is impossible to derive causal relationships, and it is thus unclear whether the activity patterns identified will affect long-term health. 

## 5. Conclusions

Associations were observed between total daily volume in MPA and VPA and more favorable cardiometabolic risk profiles. Time spent in VPA bouts was also beneficially associated with cardiometabolic risk factors, but this association was not significant after adjustment for total daily volume of VPA, indicating that total daily volume rather than accumulating this in longer bouts is most important. Detrimental associations were observed for sustained LPA and MPA bouts, which was unexpected. Perhaps prolonged behaviors may cluster with alternative unhealthy activities and/or lead to lower total daily volumes of PA, compared to sporadic behaviors. Future research should test whether sustained and sporadic behaviors are associated differently with lower total PA and explore the impact of different combinations of activities in children mostly engaging in prolonged versus sporadic PA. Research including statistically advanced analyses is required to elucidate the potential mechanisms for specific associations between different activity patterns and cardiometabolic health outcomes. Nevertheless, the results of this study suggest that activity patterns, independent of total daily volume in these intensities, could play a role in cardiometabolic health. Public health interventions and policies would benefit from further research that aims to understand how activity patterns across the activity spectrum may influence health outcomes.

## Figures and Tables

**Table 1:** Associations of total daily volume of sedentary (SED) and light- (LPA), moderate- (MPA), and vigorous-intensity physical activity (VPA) with cardiometabolic risk factors (*n* = 782).

**Minimally Adjusted Model 1**				
**Cardiometabolic risk factor**	**SED ^A^** **β (95% CI)**	**LPA ^A^** **β (95% CI)**	**MPA ^A^** **β (95% CI)**	**VPA ^A^** **β (95% CI)**
zBMI	−0.0003 (−0.0023, 0.0018)	0.0023 (−0.0004, 0.0049)	0.0015 (−0.0060, 0.0089)	−0.0168 ** (−0.0252, −0.0084)
WC	0.0067 (−0.0061, 0.0195)	0.0070 (−0.0110, 0.0249)	−0.0218 (−0.0667, 0.0231)	−0.1371 ** (−0.1898, −0.0843)
SBP ^B^	0.0061 (−0.0126, 0.0248)	−0.0067 (−0.0324, 0.0189)	0.0031 (−0.0517, 0.0578)	−0.0103 (−0.0895, 0.0690)
DBP ^B^	0.0081 (−0.0063, 0.0224)	−0.0037 (−0.0236, 0.0163)	−0.0213 (−0.0670, 0.0243)	−0.0501 (−0.1249, 0.0248)
HDL-C ^C^	−0.0004 (−0.0011, 0.0003)	−0.0003 (−0.0011, 0.0006)	0.0031 ** (0.0009, 0.0052)	0.0065 ** (0.0029, 0.0101)
LDL-C ^C^	0.0002 (−0.0011, 0.0014)	0.0010 (−0.0010, 0.0029)	−0.0029 (−0.0070, 0.0012)	−0.0079 * (−0.0145, −0.0014)
TG ^C^	0.0009 * (0.0001, 0.0017)	−0.0004 (−0.0015, 0.0006)	−0.0042 ** (−0.0063, −0.0021)	−0.0073 ** (−0.0105, −0.0041)
CMR-score ^D^	0.0056 (−0.0035, 0.0147)	0.0038 (−0.0075, 0.0151)	−0.0370 ** (−0.0609, −0.0131)	−0.0762 ** (−0.1144, −0.0380)
**Fully Adjusted Model 3**				
	**SED^A^** **β (95% CI)**	**LPA^A^** **β (95% CI)**	**MPA^A^** **β (95% CI)**	**VPA^A^** **β (95% CI)**
zBMI	0.0004 (−0.0017, 0.0025)	0.0020 (−0.0006, 0.0047)	−0.0028 (−0.0108, 0.0053)	−0.0245 ** (−0.0328, −0.0162)
WC	0.0088 (−0.0045, 0.0221)	0.0082 (−0.0100, 0.0264)	−0.0438 (−0.0918, 0.0042)	−0.1877 ** (−0.2395, −0.1359)
SBP ^B^	0.0046 (−0.0144, 0.0236)	−0.0017 (−0.0285, 0.0250)	−0.0010 (−0.0597, 0.0577)	−0.0349 (−0.1271, 0.0573)
DBP ^B^	0.0061 (−0.0081, 0.0202)	−0.0017 (−0.0221, 0.0186)	−0.0145 (−0.0650, 0.0360)	−0.0514 (−0.1390, 0.0362)
HDL-C ^C^	−0.0002 (−0.0009, 0.0005)	−0.0003 (−0.0011, 0.0005)	0.0023 * (0.0001, 0.0044)	0.0056 ** (0.0018, 0.0094)
LDL-C ^C^	0.0001 (−0.0010, 0.0013)	0.0010 (−0.0009, 0.0029)	−0.0031 (−0.0070, 0.0008)	−0.0088 * (−0.0164, −0.0013)
TG ^C^	0.0009 * (0.0002, 0.0016)	−0.0006 (−0.0016, 0.0004)	−0.0040 ** (−0.0064, −0.0016)	−0.0064 ** (−0.0103, −0.0025)
CMR-score ^D^	0.0056 (−0.0034, 0.0145)	0.0034 (−0.0075, 0.0143)	−0.0369 ** (−0.0610, −0.0128)	−0.0798 ** (−0.1205, −0.0391)

β (95% CI): regression coefficients and 95% confidence intervals. Significant findings are reported in **bold**. * (*p* < 0.05); ** (*p* < 0.01). Minimally adjusted Model 1 adjusted for wear-time and trial involvement, and accounted for clustering within schools. Fully adjusted Model 3 further adjusted for participants’ age, sex, and socioeconomic status (SES). Results from partially adjusted Model 2 (adjusted for age and sex, and not for SES, in addition to minimally adjusted Model 1) can be found in Table S4. ^A^ Variables were the average of total included valid days. ^B^ Included subset of 637 youth. ^C^ Included subset of 525 youth. ^D^ Included subset of 404 youth. SED: sedentary; LPA: light-intensity physical activity; MPA: moderate-intensity physical activity; VPA: vigorous-intensity physical activity; zBMI: body mass index converted to the World Health Organization (WHO) Child Growth Standards age- and sex-standardized z-values [20]; WC: waist circumference; SBP: systolic blood pressure; DBP: diastolic blood pressure; HDL-C: high-density lipoprotein cholesterol; LDL-C: low-density lipoprotein cholesterol; TG: triglycerides; CMR-score: cardiometabolic risk score.

## Supplementary Materials

The following are available online at https://www.mdpi.com/1660-4601/17/12/4286/s1, Figure S1: Participant flow diagram; Figure S2: Associations of median bout lengths with cardiometabolic risk factors. Table S1: Participant characteristics per trial (LOOK, Transform-Us!); Table S2: Participant characteristics of included sample, subsets, and excluded sample; Table S3: Descriptives for accelerometer-derived total daily volumes and activity patterns across the activity spectrum (*n* = 782); Table S4: Associations of total daily volume of sedentary behavior and light-, moderate-, and vigorous-intensity physical activity with cardiometabolic risk factors (*n* = 782); Table S5: Associations of activity patterns with adiposity indicators (*n* = 782) and blood pressure markers (*n* = 637); Table S6: Associations of activity patterns with lipids (*n* = 525) and cardiometabolic risk score (*n* = 404).
