# Supplementary material for: Cross-Sectional Associations of Total Daily Volume and Activity Patterns across the Activity Spectrum with Cardiometabolic Risk Factors in Children and Adolescents
Source: Int J Environ Res Public Health. 2020 Jun 16;17(12):4286. doi: 10.3390/ijerph17124286 (PMC7344989; doi:10.3390/ijerph17124286)
Supplement: Supplementary file 1 [file ijerph-17-04286-s001.pdf]

Supplementary materials

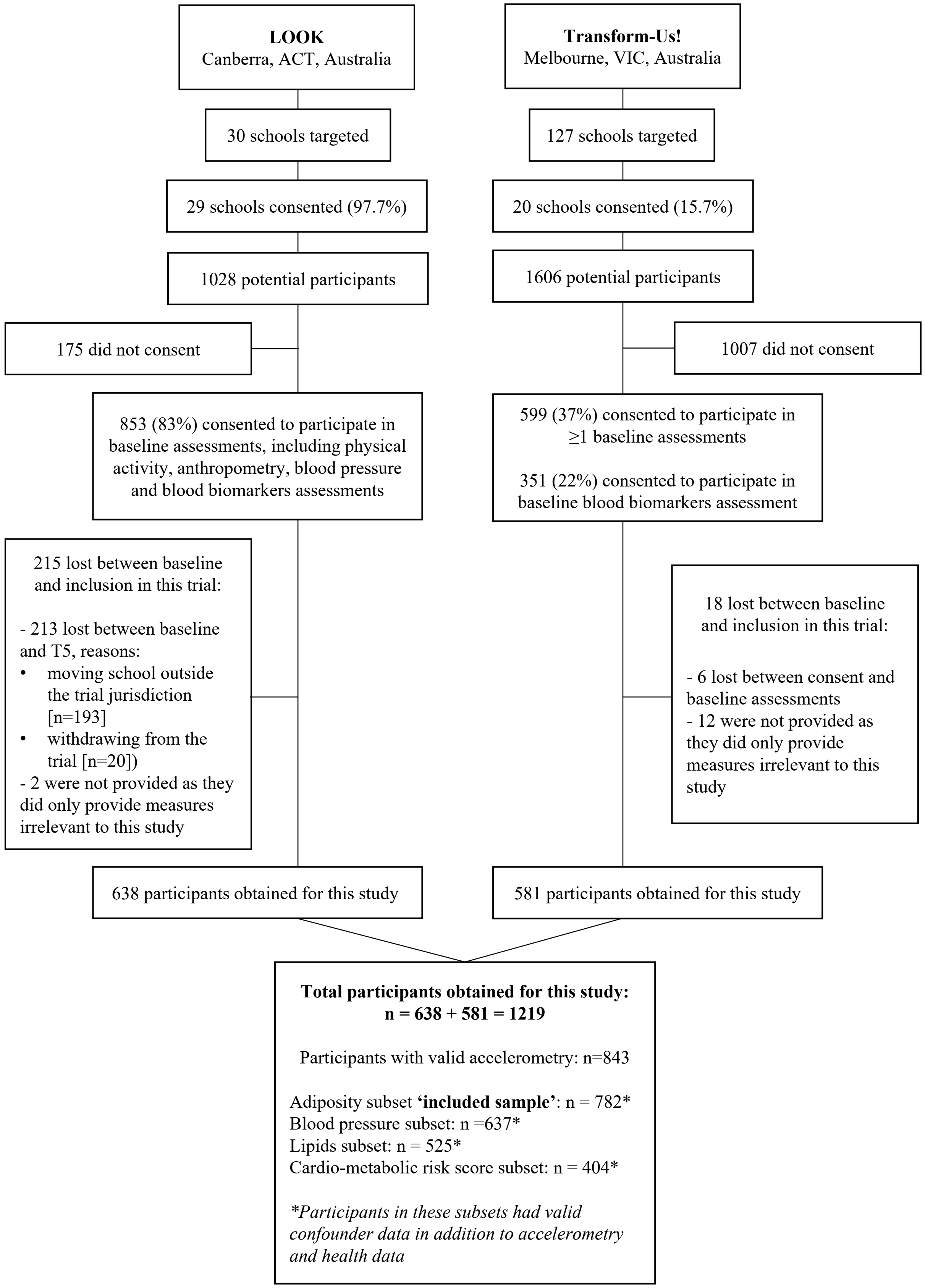

Figure S1 Participant flow diagram

Table S1. Participant characteristics per trial (LOOK, Transform-Us!)

	LOOK (n=638)[A]	Transform-Us! (n=581)[A]
**Trial commenced**	2005	2009
**Location**	Canberra, Australia	Melbourne, Australia
**Recruitment**	School-based (29/30 agreed; 97%)	School-based (20/127 agreed; 16%)
**Sampling design**	Schools were randomly allocated	Schools were randomly allocated
**Sample included**	Time-point 5 (2009)	Baseline data
**Data collection period**	Sep-Dec 2008	Feb-June 2010
**Season**	Spring/Summer	Summer/Autumn
**BMI (kg/m^2)**	19.6 ± 3.3	17.6 ± 2.9
**zBMI**	0.5 ± 1.1	0.6 ± 1.2
**WC (cm)**	67.6 ± 8.9	59.9 ± 7.1
**SBP (mmHg)**	112.4 ± 8.4	101.8 ± 8.9
**DBP (mmHg)**	61.5 ± 6.1	60.5 ± 8.5
**HDL-C (mmol/L)**	1.4 ± 0.3	1.6 ± 0.3
**LDL-C (mmol/L)**	2.5 ± 0.7	2.6 ± 0.7

**TG (mmol/L)**	0.9 ± 0.5	0.7 ± 0.3
**CMR-score**	1.6 ± 3.3	-1.8 ± 3.0
**Age (years)**	12.02 ± 0.35	8.69 ± 0.39
**Age-range (years [min-max])**	10.96-13.06	7.04-10.38

Data are presented as Mean ± SD unless otherwise indicated.

[A] These n's represent participants who had raw data for one or more assessed variables relevant to this study (total n=1219). The n's per variable in this table may vary, as not all participants had data on all of the variables (see Figure S1).

BP Blood pressure; SES Socio-Economic Status; BMI Body mass index; zBMI Body mass index converted to the World Health Organization (WHO) Child Growth Standards age and sex standardized z-values [20]; WC Waist circumference; SBP Systolic blood pressure; DBP Diastolic blood pressure; HDL-C High-density lipoprotein cholesterol; LDL-C Low-density lipoprotein cholesterol; TG Triglycerides; CMR-score Cardiometabolic risk score.

Table S2. Participant characteristics of included sample, subsets and excluded sample

	**Included sample (n=782) Mean ± SD**	**Blood pressure subset (n=637) Mean ± SD**	**Lipids subset (n=525) Mean ± SD**	**CMR-score subset (n=404) Mean ± SD**	**Excluded sample (n=437)[A] Mean ± SD**
General characteristics					
**Trial involvement**	LOOK 413 (52.8%)<br>Transform-Us! 369 (47.2%)	LOOK 279 (43.8%)<br>Transform-Us! 358 (56.2%)	LOOK 350 (66.7%)<br>Transform-Us! 175 (33.3%)	LOOK 229 (56.7%)<br>Transform-Us! 175 (43.3%)	LOOK 225 (51.5%)<br>Transform-Us! 212 (48.5%)
**Age (years)**	10.5 ± 1.7	10.2 ± 1.7	10.9 ± 1.6	10.6 ± 1.7	10.2 ± 1.7
**SES (% high/mid/low)**	3/36/61	3/33/63	1/33/65	1/30/69	7/43/49
**Sex (% female)**	55.1%	55.0%	55.1%	54.5%	50.6%
Cardiometabolic health variables					
**BMI (kg/m^2)**	18.7 ± 3.3	18.5 ± 3.3	18.9 ± 3.3	18.7 ± 3.3	18.4 ± 3.2
**zBMI**	0.5 ± 1.1	0.5 ± 1.2	0.5 ± 1.1	0.5 ± 1.1	0.5 ± 1.2

**WC (cm)**	64.1 ± 9.0	63.4 ± 9.1	65.0 ± 9.1	64.3 ± 9.3	63.1 ± 8.7
**SBP (mmHg)**	NA	106 ± 10	108 ± 10	108 ± 10	106 ± 10
**DBP (mmHg)**	NA	61 ± 8	61 ± 7	61 ± 7	61 ± 8
**HDL-C (mmol/L)**	NA	NA	1.5 ± 0.3	1.5 ± 0.3	1.5 ± 0.3
**LDL-C (mmol/L)**	NA	NA	2.5 ± 0.7	2.6 ± 0.7	2.5 ± 0.7
**TG (mmol/L)**	NA	NA	0.8 ± 0.4	0.8 ± 0.4	0.9 ± 0.6
**CMR-score**	NA	NA	NA	0.1 ± 3.6	0.3 ± 3.6

Data are presented as Mean ± SD unless otherwise indicated.

[A] Total number of excluded sample was 437 participants (i.e., participants without valid adiposity and/or accelerometry data), however, n's per variable in this table may vary, as not all participants had data on all of the variables.

BP Blood pressure; SES Socio-Economic Status; BMI Body mass index; zBMI Body mass index converted to the World Health Organization (WHO) Child Growth Standards age and sex standardized z-values [20]; WC Waist circumference; SBP Systolic blood pressure; DBP Diastolic blood pressure; HDL-C High-density lipoprotein cholesterol; LDL-C Low-density lipoprotein cholesterol; TG Triglycerides; CMR-score Cardiometabolic risk score.

Table S3. Descriptives for accelerometer-derived variables and activity patterns across the activity spectrum, of included sample (n=782)

**Variables**	**Mean ± SD**	**(% of participants engaging in behavior, if applicable)**
Accelerometer-derived variables		
Valid weekdays (no.)	4.7 ± 1.1	
Valid weekend days (no.)	1.7 ± 0.7	
Total valid days (no.)	6.3 ± 1.5	
Total wear-time (min/day)	734.1 ± 77.9	
Total daily volume SED (min/day)	437.6 ± 78.1	
Total daily volume LPA (min/day)	229.9 ± 35.4	
Total daily volume MPA (min/day)	46.0 ± 15.3	
Total daily volume VPA (min/day)	20.7 ± 12.6	
Accelerometer-derived pattern variables		
Breaks in SED time (no. per day)	310.8 ± 42.0	(100.0%)
Time accumulated in ≥5-min SED bouts (min/day)	169.6 ± 65.8	(100.0%)
Time accumulated in ≥10-min SED bouts (min/day)	81.0 ±45.9	(100.0%)
Time accumulated in ≥1-min LPA bouts (min/day)	103.9 ± 24.9	(100.0%)
Time accumulated in ≥5-min LPA bouts (min/day)	2.5 ± 3.0	(74.3%)
Time accumulated in ≥10-min LPA bouts (min/day)[A]	0.2 ± 0.9	(8.6%)
Time accumulated in ≥1-min MPA bouts (min/day)	8.6 ± 4.6	(100.0%)
Time accumulated in ≥5-min MPA bouts (min/day)[A]	0.3 ± 1.1	(17.3%)
Time accumulated in ≥10-min MPA bouts (min/day)[A]	0.0 ± 0.4	(2.2%)
Time accumulated in ≥1-min VPA bouts (min/day)	5.8 ± 6.0	(98.7%)
Time accumulated in ≥5-min VPA bouts (min/day)[A]	0.6 ± 1.9	(23.9%)
Time accumulated in ≥10-min VPA bouts (min/day)[A]	0.2 ± 1.1	(4.4%)

Breaks in SED (number per day) were defined as an interruption in SED where counts exceeded 25 counts per 15-s epoch and averaged over all valid days.

Bouts were calculated as the average daily time accumulated in either ≥1-min, ≥5-min or ≥10-min bouts of the corresponding intensities (in minutes), and averaged over included days. The presented numbers are the Mean ± SD across the sample.

[A] As only a quarter of the sample or less engaged in these activity patterns, these variables were excluded from further analyses.

SED Sedentary; LPA Light-intensity physical activity; MPA Moderate-intensity physical activity; VPA Vigorous-intensity physical activity.

Table S4. Associations of total daily volume of sedentary behavior, and in light-, moderate- and vigorous-intensity physical activity with cardiometabolic risk factors (n=782)

**Partially-adjusted Models 2**				
	SED[A] β (95% CI)	LPA[A] β (95% CI)	MPA[A] β (95% CI)	VPA[A] β (95% CI)
zBMI	0.0004 (-0.0017, 0.0025)	0.0020 (-0.0006, 0.0047)	-0.0028 (-0.0109, 0.0053)	**-0.0244** (-0.0324, -0.0163)**
WC	0.0089 (-0.0043, 0.0220)	0.0080 (-0.0100, 0.0261)	-0.0434 (-0.0914, 0.0046)	**-0.1842** (-0.2370, -0.1314)**
SBP[B]	0.0047 (-0.0139, 0.0233)	-0.0030 (-0.0287, 0.0228)	0.0012 (-0.0557, 0.0581)	-0.0243 (-0.1069, 0.0584)
DBP[B]	0.0062 (-0.0078, 0.0201)	-0.0022 (-0.0223, 0.0180)	-0.0137 (-0.0632, 0.0358)	-0.0471 (-0.1303, 0.0361)
HDL-C[C]	-0.0002 (-0.0009, 0.0005)	-0.0003 (-0.0011, 0.0005)	**0.0022* (0.0001, 0.0043)**	**0.0054** (0.0017, 0.0092)**
LDL-C[C]	0.0002 (-0.0010, 0.0013)	0.0010 (-0.0010, 0.0029)	-0.0030 (-0.0069, 0.0008)	**-0.0085* (-0.0160, -0.0010)**
TG[D]	**0.0009* (0.0002, 0.0016)**	-0.0006 (-0.0016, 0.0004)	**-0.0040** (-0.0063, -0.0016)**	**-0.0062** (-0.0100, -0.0024)**
CMR-score[D]	0.0051 (-0.0038, 0.0140)	0.0038 (-0.0072, 0.0149)	**-0.0363** (-0.0602, -0.0124)**	**-0.0771** (-0.1183, -0.0358)**

β (95% CI) Regression coefficients and 95% confidence intervals. Significant findings are reported in **bold**.

* (p < 0.05); ** (p < 0.01).

Partially-adjusted Model 2 adjusted for participants' wear-time and trial involvement, age, sex, and accounted for clustering within schools. Minimally-adjusted Model 1 and fully-adjusted Model 3 can be found in Table 1 within the manuscript.

A Variables were averaged of total included valid day.

B Included subset of 637 youth.

C Included subset of 525 youth.

D Included subset of 404 youth.

SED Sedentary; LPA Light-intensity physical activity; MPA Moderate-intensity physical activity; MVPA Moderate-to-vigorous-intensity physical activity; VPA Vigorous-intensity physical activity; zBMI Body mass index converted to the World Health Organization (WHO) Child Growth Standards age and sex standardized z-values [20]; WC Waist circumference; SBP Systolic blood pressure; DBP Diastolic blood pressure; HDL-C High-density lipoprotein cholesterol; LDL-C Low-density lipoprotein cholesterol; TG Triglycerides; CMR-score Cardiometabolic risk score.

Table S5. Associations of activity patterns with adiposity indicators (n=782) and blood pressure markers (n=637)

		zBMI	Waist circumference	Systolic blood pressure	Diastolic blood pressure
		β (95% CI)	β (95% CI)	β (95% CI)	β (95% CI)
**Breaks in SED time**	Model 1	-0.0016 (-0.0046, 0.0014)	-0.0127 (-0.0366, 0.0113)	-0.0222 (-0.0486, 0.0043)	0.0043 (-0.0162, 0.0248)
**(no. per day)**	Model 2	-0.0016 (-0.0046, 0.0014)	-0.0128 (-0.0367, 0.0112)	-0.0222 (-0.0486, 0.0043)	0.0043 (-0.0160, 0.0246)
	Model 3	-0.0007 (-0.0040, 0.0025)	-0.0053 (-0.0295, 0.0190)	-0.0173 (-0.0436, 0.0090)	0.0044 (-0.0193, 0.0281)
	Model 4	-0.0007 (-0.0039, 0.0025)	-0.0049 (-0.0292, 0.0195)	-0.0157 (-0.0414, 0.0101)	0.0051 (-0.0185, 0.0288)
**SED ≥5-min bouts**	Model 1	0.0016 (-0.0017, 0.0049)	0.0077 (-0.0206, 0.0361)	0.0222 (-0.0088, 0.0532)	-0.0047 (-0.0275, 0.0180)
**(min/day)**	Model 2	0.0016 (-0.0017, 0.0050)	0.0075 (-0.0208, 0.0358)	0.0200 (-0.0103, 0.0503)	-0.0050 (-0.0276, 0.0176)
	Model 3	0.0009 (-0.0025, 0.0043)	0.0024 (-0.0252, 0.0300)	0.0184 (-0.0106, 0.0474)	-0.0040 (-0.0279, 0.0199)
	Model 4	0.0008 (-0.0026, 0.0043)	0.0020 (-0.0256, 0.0296)	0.0220 (-0.0090, 0.0530)	-0.0049 (-0.0281, 0.0184)
**SED ≥10-min bouts**	Model 1	0.0006 (-0.0029, 0.0040)	-0.0011 (-0.0293, 0.0271)	0.0276 (-0.0055, 0.0607)	-0.0077 (-0.0330, 0.0175)
**(min/day)**	Model 2	0.0006 (-0.0029, 0.0040)	-0.0015 (-0.0296, 0.0265)	0.0273 (-0.0056, 0.0602)	-0.0082 (-0.0332, 0.0167)

	Model 3	-0.0001 (-0.0036, 0.0035)	-0.0051 (-0.0328, 0.0227)	0.0267 (-0.0061, 0.0595)	-0.0071 (-0.0329, 0.0188)
	Model 4	-0.0001 (-0.0037, 0.0034)	-0.0059 (-0.0333, 0.0216)	0.0246 (-0.0064, 0.0557)	-0.0084 (-0.0329, 0.0161)
**LPA ≥1-min bouts**	Model 1	**0.0063** (0.0026, 0.0099)**	**0.0375** (0.0142, 0.0608)**	0.0081 (-0.0264, 0.0426)	-0.0011 (-0.0296, 0.0275)
**(min/day)**	Model 2	**0.0247** (0.0162, 0.0333)**	**0.2091** (0.1451, 0.2731)**	**0.1122* (0.0223, 0.2021)**	0.0239 (-0.0336, 0.0815)
	Model 3	**0.0264** (0.0178, 0.0351)**	**0.2091** (0.1449, 0.2733)**	**0.1035* (0.0134, 0.1937)**	0.0182 (-0.0367, 0.0732)
	Model 4	**0.0264** (0.0177, 0.0351)**	**0.2097** (0.1460, 0.2735)**	**0.1065* (0.0113, 0.2017)**	0.0182 (-0.0376, 0.0741)
**LPA ≥5-min bouts**	Model 1	**0.0532** (0.0269, 0.0794)**	**0.4169** (0.1895, 0.6443)**	0.1214 (-0.1351, 0.3780)	0.1613 (-0.0638, 0.3863)
**(min/day)**	Model 2	**0.0514** (0.0254, 0.0773)**	**0.4599** (0.2173, 0.7025)**	0.1738 (-0.1081, 0.4557)	0.2056 (-0.0301, 0.4413)
	Model 3	**0.0518** (0.0257, 0.0779)**	**0.4291** (0.1859, 0.6722)**	0.1158 (-0.1704, 0.4019)	0.1952 (-0.0247, 0.4150)
	Model 4	**0.0519** (0.0257, 0.0781)**	**0.4290** (0.1851, 0.6728)**	0.1213 (-0.1748, 0.4174)	0.1981 (-0.0235, 0.4197)
**MPA ≥1-min bouts**	Model 1	**0.0333** (0.0134, 0.0532)**	**0.1657* (0.0099, 0.3215)**	0.1403 (-0.0123, 0.2928)	0.0588 (-0.0659, 0.1834)
**(min/day)**	Model 2	**0.0603** (0.0349, 0.0857)**	**0.4077** (0.1814, 0.6340)**	**0.2668* (0.0211, 0.5125)**	0.1961 (-0.0412, 0.4333)
	Model 3	**0.0619** (0.0378, 0.0860)**	**0.4065** (0.1908, 0.6222)**	**0.2525* (0.0127, 0.4923)**	0.1921 (-0.0415, 0.4257)

	Model 4	**0.0621** (0.0379, 0.0863)**	**0.4085** (0.1922, 0.6247)**	**0.2589* (0.0208, 0.4971)**	0.1952 (-0.0388, 0.4291)
**VPA ≥1-min bouts**	Model 1	**-0.0259** (-0.0369, -0.0149)**	**-0.1717** (-0.2646, -0.0788)**	**-0.0930* (-0.1706, -0.0154)**	**-0.0888* (-0.1758, -0.0018)**
**(min/day)**	Model 2	-0.0184 (-0.0378, 0.0009)	-0.0622 (-0.2344, 0.1101)	**-0.1955** (-0.3196, -0.0713)**	-0.0804 (-0.2202, 0.0594)
	Model 3	-0.0112 (-0.0314, 0.0089)	-0.0181 (-0.2051, 0.1689)	**-0.1784** (-0.3107, -0.0460)**	-0.0835 (-0.2315, 0.0645)
	Model 4	-0.0110 (-0.0312, 0.0093)	-0.0135 (-0.1998, 0.1728)	**-0.1691* (-0.3087, -0.0296)**	-0.0793 (-0.2274, 0.0689)
**Median SED bouts**	Model 1	0.0166 (-0.1055, 0.1387)	0.1085 (-0.8204, 1.0374)	0.6906 (-0.4506, 1.8318)	-0.0164 (-1.0074, 0.9745)
**(min)**	Model 2	0.0255 (-0.1078, 0.1588)	-0.0393 (-1.0448, 0.9662)	0.6439 (-0.5470, 1.8349)	-0.2132 (-1.3452, 0.9188)
	Model 3	0.0127 (-0.1208, 0.1463)	-0.0806 (-1.0818, 0.9205)	0.6811 (-0.5190, 1.8812)	-0.1737 (-1.3244, 0.9769)
	Model 4	0.0077 (-0.1243, 0.1397)	-0.1290 (-1.0943, 0.8362)	0.6015 (-0.5376, 1.7407)	-0.2344 (-1.3202, 0.8515)
**Median LPA bouts**	Model 1	**2.8998* (0.6493, 5.1504)**	**26.3984** (11.1481, 41.6488)**	-2.6662 (-19.0809, 13.7486)	5.1342 (-4.0340, 14.3025)
**(min)**	Model 2	**2.7182* (0.4122, 5.0241)**	**28.8048** (11.4550, 46.1545)**	-1.1139 (-18.6227, 16.3949)	7.2014 (-4.5408, 18.9435)
	Model 3	**2.7861* (0.4584, 5.1139)**	**27.5975** (10.2718, 44.9231)**	-3.2535 (-20.5596, 14.0525)	6.5046 (-4.6497, 17.6590)
	Model 4	**2.7891* (0.4591, 5.1192)**	**27.4903** (10.1654, 44.8152)**	-4.0566 (-21.9370, 13.8239)	6.2432 (-4.8003, 17.2866)

**Median MPA bouts**	Model 1	0.2402 (-0.1993, 0.6798)	1.1875 (-2.7352, 5.1102)	1.0126 (-3.3381, 5.3633)	0.1477 (-3.4475, 3.7429)
**(min)**	Model 2	0.2295 (-0.2212, 0.6801)	1.5477 (-2.5806, 5.6760)	1.0037 (-3.6151, 5.6226)	0.4417 (-3.1953, 4.0787)
	Model 3	0.2957 (-0.1606, 0.7520)	1.8449 (-2.2367, 5.9266)	1.1574 (-3.5121, 5.8269)	0.3560 (-3.3776, 4.0896)
	Model 4	0.2991 (-0.1492, 0.7474)	1.9338 (-2.0764, 5.9439)	1.4889 (-3.1879, 6.1656)	0.5020 (-3.2266, 4.2305)
**Median VPA bouts**	Model 1	**-0.3234** (-0.5000, -0.1469)**	**-2.1883* (-3.8404, -0.5361)**	-0.4419 (-1.6943, 0.8105)	-0.6768 (-1.8159, 0.4623)
**(min)**	Model 2	**-0.2086* (-0.4062, -0.0111)**	-1.1168 (-2.9712, 0.7375)	-0.4313 (-1.8428, 0.9802)	-0.1864 (-1.5004, 1.1275)
	Model 3	-0.1876 (-0.3806, 0.0053)	-0.9069 (-2.7503, 0.9365)	-0.2746 (-1.6986, 1.1494)	-0.1703 (-1.4956, 1.1550)
	Model 4	-0.1860 (-0.3800, 0.0081)	-0.8374 (-2.6957, 1.0208)	-0.1187 (-1.5477, 1.3102)	-0.1058 (-1.3917, 1.1802)

β (95% CI) Regression coefficients and 95% confidence intervals. Significant findings are reported in **bold**.

* ($p < 0.05$); ** ($p < 0.01$).

Minimally-adjusted Model 1 adjusted for wear-time and trial involvement, and accounted for clustering within schools. Partially-adjusted Model 2 additionally adjusted for total daily volume of the corresponding intensity. Partially-adjusted Model 3 additionally adjusted for participants' age and sex, and fully-adjusted Model 4 further adjusted for socio-economic status.

Breaks in SED (number per day) were defined as an interruption in SED where counts exceeded 25 counts per 15-s epoch and averaged over all valid days.

Bouts were calculated as the average daily time accumulated in either ≥1-min, ≥5-min or ≥10-min bouts of the corresponding intensities (in minutes), and averaged over included days. The presented numbers are the Mean ± SD across the sample.

SED Sedentary; LPA Light-intensity physical activity; MPA Moderate-intensity physical activity; VPA Vigorous-intensity physical activity; zBMI Body mass index converted to the World Health Organization (WHO) Child Growth Standards age and sex standardized z-values [20].

Table S6. Associations of activity patterns with lipids (n=525) and cardiometabolic risk score (n=404)

		HDL-C	LDL-C	Triglycerides	CMR-score
		**β (95% CI)**	**β (95% CI)**	**β (95% CI)**	**β (95% CI)**
**Breaks in SED time**	Model 1	-0.0007 (-0.0017, 0.0002)	0.0008 (-0.0015, 0.0031)	0.0004 (-0.0007, 0.0015)	0.0066 (-0.0047, 0.0179)
**(no. per day)**	Model 2	-0.0007 (-0.0017, 0.0002)	0.0008 (-0.0015, 0.0031)	0.0004 (-0.0007, 0.0015)	0.0069 (-0.0041, 0.0178)
	Model 3	-0.0004 (-0.0013, 0.0006)	0.0008 (-0.0017, 0.0032)	-0.0001 (-0.0014, 0.0011)	0.0058 (-0.0054, 0.0171)
	Model 4	-0.0004 (-0.0013, 0.0006)	0.0008 (-0.0017, 0.0033)	-0.0001 (-0.0014, 0.0012)	0.0057 (-0.0055, 0.0169)
**SED ≥5-min bouts**	Model 1	0.0002 (-0.0008, 0.0012)	-0.0001 (-0.0027, 0.0024)	0.0004 (-0.0009, 0.0017)	-0.0008 (-0.0142, 0.0127)
**(min/day)**	Model 2	0.0002 (-0.0007, 0.0012)	-0.0001 (-0.0026, 0.0024)	0.0004 (-0.0009, 0.0016)	-0.0012 (-0.0145, 0.0121)
	Model 3	-0.0000 (-0.0010, 0.0010)	-0.0001 (-0.0026, 0.0025)	0.0006 (-0.0007, 0.0019)	-0.0002 (-0.0135, 0.0130)
	Model 4	-0.0000 (-0.0010, 0.0010)	-0.0001 (-0.0027, 0.0025)	0.0006 (-0.0007, 0.0020)	-0.0002 (-0.0135, 0.0131)
**SED ≥10-min bouts**	Model 1	0.0001 (-0.0010, 0.0012)	0.0002 (-0.0026, 0.0030)	0.0007 (-0.0006, 0.0020)	0.0014 (-0.0134, 0.0161)
**(min/day)**	Model 2	0.0002 (-0.0009, 0.0012)	0.0002 (-0.0026, 0.0029)	0.0006 (-0.0006, 0.0019)	0.0011 (-0.0134, 0.0157)

	Model 3	-0.0000 (-0.0010, 0.0010)	0.0002 (-0.0026, 0.0030)	0.0007 (-0.0006, 0.0021)	0.0019 (-0.0126, 0.0163)
	Model 4	-0.0000 (-0.0010, 0.0010)	0.0002 (-0.0026, 0.0030)	0.0007 (-0.0006, 0.0020)	0.0014 (-0.0128, 0.0157)
**LPA ≥1-min bouts**	Model 1	-0.0009 (-0.0020, 0.0002)	0.0022 (-0.0005, 0.0049)	0.0002 (-0.0013, 0.0017)	**0.0155* (0.0018, 0.0291)**
**(min/day)**	Model 2	**-0.0042* (-0.0074, -0.0009)**	0.0072 (-0.0003, 0.0146)	**0.0058** (0.0022, 0.0094)**	**0.0719** (0.0352, 0.1087)**
	Model 3	**-0.0042* (-0.0074, -0.0010)**	**0.0073* (0.0000, 0.0145)**	**0.0064** (0.0028, 0.0100)**	**0.0719** (0.0354, 0.1084)**
	Model 4	**-0.0042* (-0.0074, -0.0010)**	**0.0074* (0.0001, 0.0147)**	**0.0065** (0.0029, 0.0101)**	**0.0727** (0.0360, 0.1093)**
**LPA ≥5-min bouts**	Model 1	**-0.0144** (-0.0217, -0.0070)**	0.0073 (-0.0176, 0.0321)	0.0092 (-0.0067, 0.0250)	**0.1755** (0.0525, 0.2984)**
**(min/day)**	Model 2	**-0.0155** (-0.0244, -0.0066)**	0.0034 (-0.0238, 0.0306)	0.0133 (-0.0030, 0.0295)	**0.1812* (0.0337, 0.3286)**
	Model 3	**-0.0170** (-0.0259, -0.0082)**	0.0039 (-0.0232, 0.0310)	**0.0173* (0.0014, 0.0331)**	**0.1879* (0.0374, 0.3385)**
	Model 4	**-0.0168** (-0.0257, -0.0079)**	0.0034 (-0.0235, 0.0302)	**0.0170* (0.0009, 0.0331)**	**0.1885* (0.0375, 0.3395)**
**MPA ≥1-min bouts**	Model 1	0.0024 (-0.0026, 0.0075)	-0.0043 (-0.0153, 0.0067)	-0.0028 (-0.0083, 0.0028)	-0.0116 (-0.0786, 0.0553)
**(min/day)**	Model 2	**-0.0064* (-0.0125, -0.0003)**	0.0019 (-0.0155, 0.0194)	0.0101 (-0.0004, 0.0206)	**0.1195* (0.0016, 0.2374)**
	Model 3	**-0.0061* (-0.0122, -0.0000)**	0.0021 (-0.0154, 0.0196)	**0.0105* (0.0003, 0.0207)**	**0.1199* (0.0014, 0.2383)**

	Model 4	-0.0060 (-0.0121, 0.0000)	0.0020 (-0.0155, 0.0196)	**0.0103* (0.0002, 0.0205)**	**0.1252* (0.0067, 0.2436)**
**VPA ≥1-min bouts**	Model 1	0.0035 (-0.0015, 0.0086)	**-0.0102* (-0.0191, -0.0013)**	-0.0030 (-0.0078, 0.0018)	**-0.0703** (-0.1127, -0.0280)**
**(min/day)**	Model 2	**-0.0056* (-0.0107, -0.0006)**	-0.0054 (-0.0217, 0.0110)	**0.0083* (0.0010, 0.0157)**	0.0148 (-0.0375, 0.0671)
	Model 3	-0.0048 (-0.0099, 0.0003)	-0.0050 (-0.0213, 0.0113)	**0.0079* (0.0008, 0.0149)**	0.0164 (-0.0395, 0.0724)
	Model 4	-0.0052 (-0.0106, 0.0001)	-0.0043 (-0.0207, 0.0121)	**0.0084* (0.0008, 0.0160)**	0.0192 (-0.0385, 0.0769)
**Median SED bouts**	Model 1	-0.0065 (-0.0578, 0.0447)	0.0241 (-0.0898, 0.1379)	0.0294 (-0.0266, 0.0854)	0.3164 (-0.2881, 0.9209)
**(min)**	Model 2	0.0019 (-0.0466, 0.0503)	0.0226 (-0.0828, 0.1281)	0.0100 (-0.0430, 0.0629)	0.2177 (-0.3502, 0.7856)
	Model 3	-0.0010 (-0.0470, 0.0450)	0.0229 (-0.0840, 0.1298)	0.0091 (-0.0422, 0.0603)	0.2296 (-0.3337, 0.7929)
	Model 4	-0.0008 (-0.0478, 0.0463)	0.0216 (-0.0848, 0.1279)	0.0092 (-0.0419, 0.0603)	0.1913 (-0.3530, 0.7356)
**Median LPA bouts**	Model 1	**-0.6084* (-1.1361, -0.0806)**	0.8721 (-0.4870, 2.2311)	0.6065 (-0.3393, 1.5523)	7.2704 (-0.5801, 15.1208)
**(min)**	Model 2	**-0.6108* (-1.2210, -0.0005)**	0.7248 (-0.6886, 2.1381)	0.7983 (-0.2307, 1.8272)	7.1844 (-2.2748, 16.6436)
	Model 3	**-0.6659* (-1.2777, -0.0541)**	0.7464 (-0.6381, 2.1309)	0.9349 (-0.0525, 1.9224)	7.4014 (-2.0341, 16.8370)
	Model 4	**-0.6638* (-1.2751, -0.0525)**	0.7358 (-0.6632, 2.1349)	0.9341 (-0.0538, 1.9219)	7.2404 (-2.2422, 16.7231)

**Median MPA bouts**	Model 1	-0.0379 (-0.2036, 0.1278)	-0.0884 (-0.4550, 0.2782)	0.2172 (-0.0685, 0.5028)	0.5524 (-2.0407, 3.1455)
**(min)**	Model 2	-0.0830 (-0.2591, 0.0932)	-0.0518 (-0.4465, 0.3429)	0.2869 (-0.0151, 0.5889)	1.0930 (-1.6413, 3.8273)
	Model 3	-0.0679 (-0.2405, 0.1047)	-0.0501 (-0.4454, 0.3453)	0.2797 (-0.0125, 0.5719)	1.0768 (-1.6639, 3.8176)
	Model 4	-0.0732 (-0.2421, 0.0957)	-0.0402 (-0.4417, 0.3613)	0.2872 (-0.0016, 0.5759)	1.1241 (-1.5994, 3.8476)
**Median VPA bouts**	Model 1	**0.0649* (0.0088, 0.1211)**	-0.1446 (-0.3008, 0.0117)	-0.0423 (-0.1463, 0.0617)	-0.7885 (-1.5869, 0.0099)
**(min)**	Model 2	0.0039 (-0.0659, 0.0737)	-0.0914 (-0.2800, 0.0972)	0.0362 (-0.0802, 0.1525)	0.0886 (-0.7225, 0.8997)
	Model 3	0.0070 (-0.0631, 0.0771)	-0.0918 (-0.2775, 0.0939)	0.0247 (-0.0864, 0.1358)	0.0954 (-0.7193, 0.9100)
	Model 4	-0.0003 (-0.0692, 0.0686)	-0.0796 (-0.2662, 0.1070)	0.0347 (-0.0824, 0.1519)	0.1486 (-0.6750, 0.9722)

β (95% CI) Regression coefficients and 95% confidence intervals. Significant findings are reported in **bold**.

* ($p < 0.05$); ** ($p < 0.01$).

Minimally-adjusted Model 1 adjusted for wear-time and trial involvement, and accounted for clustering within schools. Partially-adjusted Model 2 additionally adjusted for total daily volume of the corresponding intensity. Partially-adjusted Model 3 additionally adjusted for participants' age and sex, and fully-adjusted Model 4 further adjusted for socio-economic status.

Breaks in SED (number per day) were defined as an interruption in SED where counts exceeded 25 counts per 15-s epoch and averaged over all valid days.

Bouts were calculated as the average daily time accumulated in either ≥1-min, ≥5-min or ≥10-min bouts of the corresponding intensities (in minutes), and averaged over included days. The presented numbers are the Mean ± SD across the sample.

SED Sedentary; LPA Light-intensity physical activity; MPA Moderate-intensity physical activity; VPA Vigorous-intensity physical activity; HDL-C High-density lipoprotein cholesterol; LDL-C Low-density lipoprotein cholesterol; CMR-score Cardiometabolic risk score.

Figure S2a. zBMI (n=782)

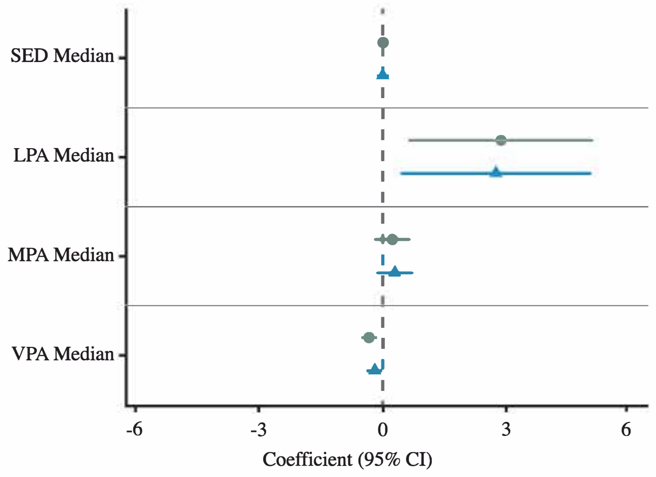

Figure S2b. Waist circumference (n=782)

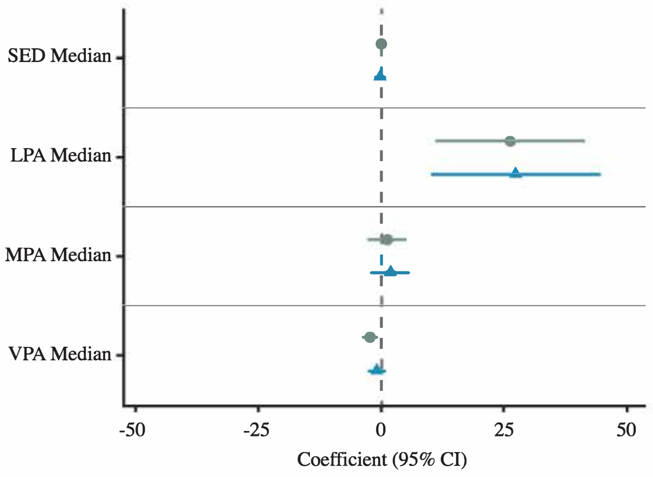

Figure S2c. Systolic blood pressure (n=637)

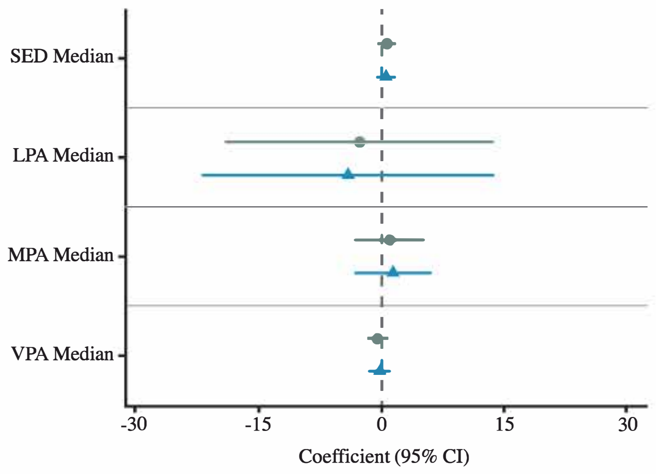

Figure S2d. Diastolic blood pressure (n=637)

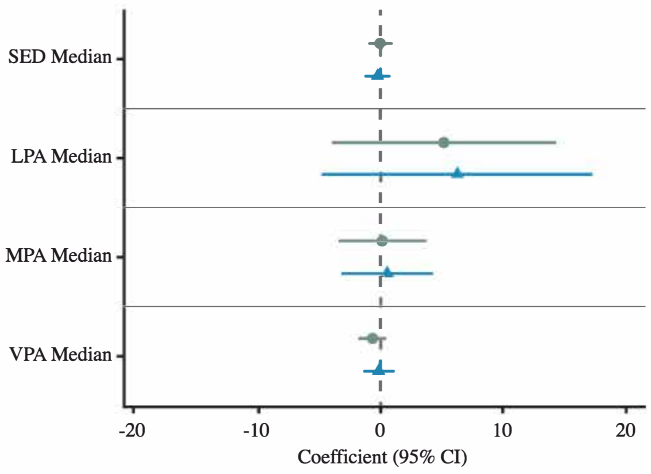

Figure S2e. High-density lipoprotein cholesterol (n=525)

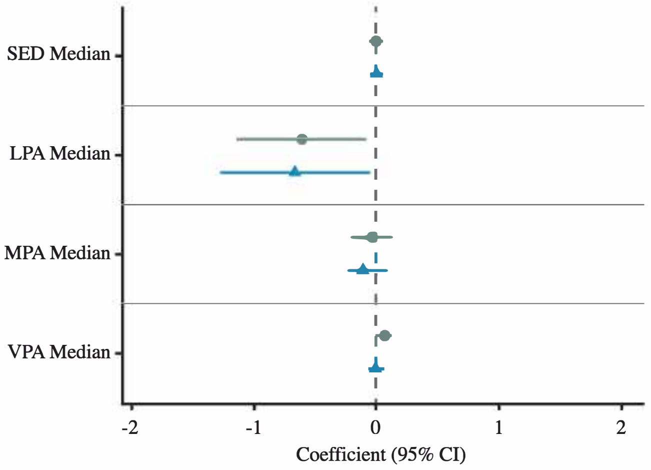

Figure S2f. Low-density lipoprotein cholesterol (n=525)

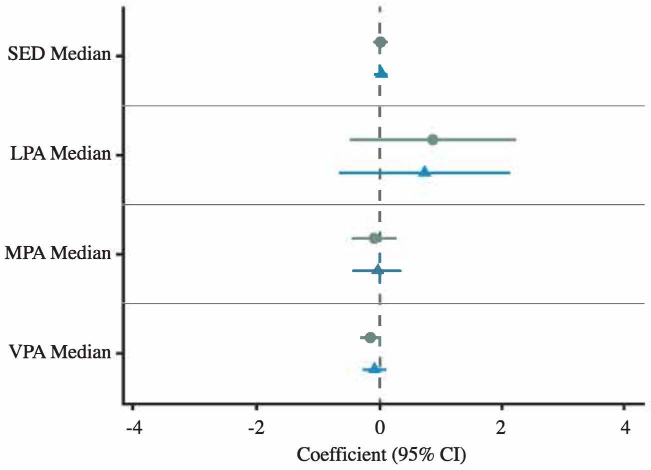

Figure S2g. Triglycerides (n=525)

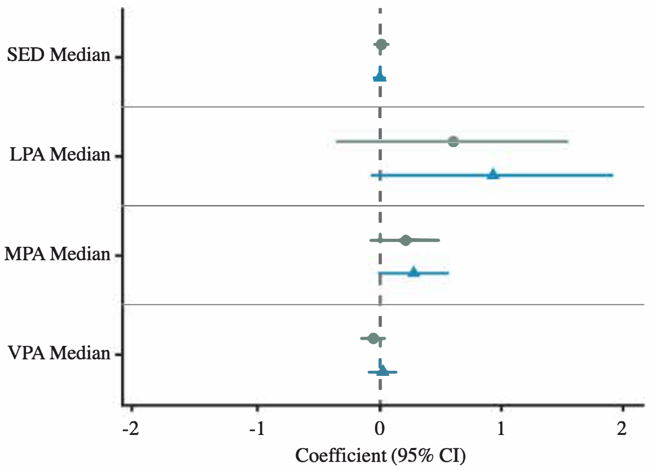

Figure S2h. Cardiometabolic risk score (n=404)

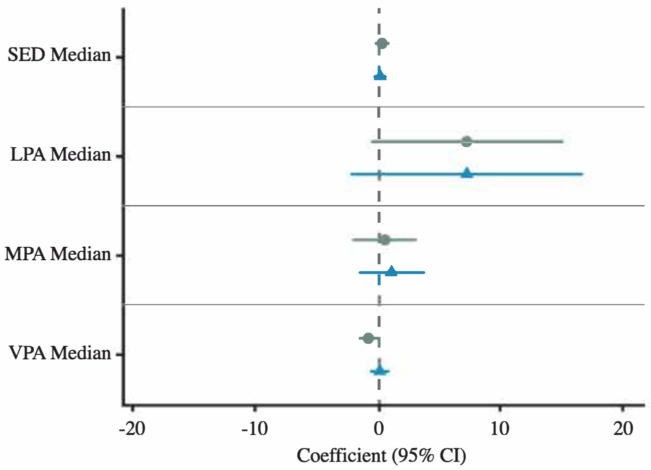

Figure S2. Associations of median bout lengths with cardiometabolic risk factors

Coefficient (95% CI) Regression coefficients and 95% confidence intervals. Coefficients were standardised across figures to aid comparison between variables.

● Model 1 (adjusted for wear-time and trial involvement, and accounted for clustering within schools).

▲ Model 4 (further adjusted for the total daily volume of the corresponding intensity, participants' age, sex, and socio-economic status).

Results from the partially-adjusted Models 2-3 can be found in Tables S5-S6.

Median bout durations (in minutes) for all intensities were estimated by calculating all uninterrupted shortest bouts within the same intensity from smallest to largest and then identifying the mid-point [18].

SED Sedentary; *LPA* Light-intensity physical activity; *MPA* Moderate-intensity physical activity; *VPA* Vigorous-intensity physical activity; z*BMI* Body mass index converted to the World Health Organization (WHO) Child Growth Standards age and sex standardized z-values [20]; *min* Minutes.
